# Catalytic Oxidation Activity of NO over Mullite-Supported Amorphous Manganese Oxide Catalyst

**DOI:** 10.3390/ma16103821

**Published:** 2023-05-18

**Authors:** Jianlin Yang, Lu Zhao, Tianran Zhou, Shuhua Ma, Xiaohui Wang

**Affiliations:** 1 College of Materials Science and Engineering, Liaoning Technical University, Fuxin 123000, China; zhaol0304@163.com; 2 School of Civil Engineering, Liaoning Technical University, Fuxin 123000, China; 3 CAS Key Laboratory for Green Processes and Engineering, Institute of Process Engineering, Chinese Academy of Sciences, Beijing 100190, China; 4 Innovation Academy for Green Manufacture, Chinese Academy of Sciences, Beijing 100190, China; 5 National Engineering Research Center of Green Recycling for Strategic Metal Resources, Institute of Process Engineering, Chinese Academy of Sciences, Beijing 100190, China

**Keywords:** high-alumina coal fly ash, mullite, nitric oxide, catalytic oxidation, manganese oxide

## Abstract

Nitric oxide (NO) can pose a severe threat to human health and the environment. Many catalytic materials that contain noble metals can oxidize NO into NO_2_. Therefore, the development of a low-cost, earth-abundant, and high-performance catalytic material is essential for NO removal. In this study, mullite whiskers on a micro-scale spherical aggregate support were obtained from high-alumina coal fly ash using an acid–alkali combined extraction method. Microspherical aggregates and Mn(NO_3_)_2_ were used as the catalyst support and the precursor, respectively. A mullite-supported amorphous manganese oxide (MSAMO) catalyst was prepared by impregnation and calcination at low temperatures, in which amorphous MnO_x_ is evenly dispersed on the surface and inside of aggregated microsphere support. The MSAMO catalyst, with a hierarchical porous structure, exhibits high catalytic performance for the oxidation of NO. The MSAMO catalyst, with a 5 wt% MnO_x_ loading, presented satisfactory NO catalytic oxidation activity at 250 °C, with an NO conversion rate as high as 88%. Manganese exists in a mixed-valence state in amorphous MnO_x_, and Mn^4+^ provides the main active sites. The lattice oxygen and chemisorbed oxygen in amorphous MnO_x_ participate in the catalytic oxidation of NO into NO_2_. This study provides insights into the effectiveness of catalytic NO removal in practical industrial coal-fired boiler flue gas. The development of high-performance MSAMO catalysts represents an important step towards the production of low-cost, earth-abundant, and easily synthesized catalytic oxidation materials.

## 1. Introduction

Nitric oxide (NO) is produced during high-temperature fossil-fuel combustion and leads to photochemical smog, acid rain, and ozone depletion, all of which are severely harmful to the environment and human health [1,2]. Therefore, reducing NO emissions from fossil fuels is essential to address climate change concerns. One of the crucial steps in NO removal is the oxidation of NO to NO_2_. Selective catalytic oxidation (SCO) is regarded as a key step for NO removal, which is conducive to improve the catalytic activity of catalysts. When the SCO method is combined with wet absorption by alkaline solution, it can achieve good denitration efficiency [3,4], aiding in nitrogen recovery and utilization. The SCO method has excellent prospects for application in the industry, due to its benefits of non-additional oxidants, low cost, and low secondary pollution. Generally, the catalytic oxidation efficiency of NO is low. To improve the efficiency, two key challenges must be addressed: enhancing the intrinsic activity of catalysts and increasing the contact area between the catalysts and NO [5]. Consequently, researchers are focusing on developing suitable catalytic materials for NO oxidation.

Noble metal catalysts, including platinum (Pt), gold (Au), and ruthenium (Ru) [6,7,8], exhibit superior catalytic activity and resistance to high temperatures, making them popular in NO catalysis [9]. They currently remain the best material option at approximately 250 °C, while their catalytic performance is affected by the support, dispersion, loading, oxide formation, and calcination temperature. Moreover, their high cost limits any large-scale industrial application. In recent years, transition metal oxides, such as manganese (Mn)-based, cobalt (Co)-based, and iron (Fe)-based catalysts, have become the research focus because of their high catalytic activity, low cost, and high yield [10,11,12]. Among these oxides, MnO_x_ is considered one of the most efficient catalysts because of the Mn^4+^/Mn^3+^ or Mn^3+^/Mn^2+^ redox cycle related to the multivalence Mn ions, structural diversity, and good oxygen mobility. Given its excellent redox ability at low temperatures, it has received considerable attention as a potential NO oxidation catalyst [13]. Manganese ions are the reactive sites for NO oxidation, with Mn^4+^ exhibiting the best activity [14,15]. The crystallographic structures of MnO_2_ play a crucial role in catalytic oxidation activity [16]. Considerable differences in the activity of MnO_2_ have been observed depending on its structures. Among the four crystallographic forms (α, β, γ, and δ), γ-MnO_2_ exhibited the best activity, converting of more than 80% of NO at 250 °C [17]. Moreover, the chemisorbed oxygen and lattice oxygen of MnO_x_ promoted the NO oxidation process, and the chemisorbed oxygen could boost the adsorption of NO on MnO_x_ surface [18]. The further development of the oxidation property of catalysts was still desired. The introduction of oxides as the dopants could considerably enhance the catalytic performance of MnO_x_ due to the formation of a solid solution, which could enhance the mobility of oxygen [19]. Compared to crystal phase of transition metal oxides, the amorphous phase exhibits improved physicochemical properties, such as high catalytic activity and durability [20]. Amorphous MnO_x_ potentially has higher NO catalytic efficiency because of its uniform active oxygen species distributions and the presence of abundant unsaturated atoms on its surface. However, the fabrication of an amorphous MnO_x_ catalyst is still challenging.

The oxidation rate of NO is primarily affected by the catalyst support, which is more important than other factors such as calcination temperature, calcination atmosphere, pretreatment, and precursor material [21]. The contact surface area between the catalyst and NO can be increased by the catalyst support, thus reducing the required quantity of the catalyst. Therefore, the supported metal oxide catalysts have received growing attention due to the low cost, high catalytic activity and stability. However, the cost of catalyst support is still high [22]. It is essential to develop catalyst supports that exhibit low cost and excellent performance. Mullite is an ideal support for the catalytic oxidation of NO due to its excellent chemical and thermal stability, as well as its high mechanical strength [23]. Its wide aperture can effectively reduce the diffusion resistance between the molecules and prolong the service life of the catalyst.

High-alumina coal fly ash (HACFA), a characteristic secondary resource available in China, contains 40–50 wt% Al_2_O_3_ and mainly consists of crystalline mullite, amorphous SiO_2_, and amorphous Al_2_O_3_. Mullite whiskers in HACFA are coated with amorphous SiO_2_ and Al_2_O_3_, forming a core-shell structure [24]. Consequently, HACFA is a promising material for mullite support. However, there were few studies focusing on mullite whiskers and amorphous MnO_x_. In this study, mullite whisker micro-scale spherical aggregates (MA), which were used as catalyst support, were obtained using the acid–alkali combined extraction method to remove the amorphous phase from HACFA. Mullite-supported amorphous manganese oxide (MSAMO) catalysts for the SCO reaction of NO were prepared via a simple impregnation and calcination method, in which amorphous MnO_x_ was loaded into MA using Mn(NO_3_)_2_ as the precursor. The microstructural characteristics of MSAMO were investigated, and the catalytic oxidation activity of NO over MSAMO was evaluated. In situ diffuse reflectance infrared Fourier transform spectroscopy (DRIFTS) was implemented to clarify the catalytic oxidation mechanisms of NO over MSAMO. This work contributes to the development of a low-cost, earth-abundant, and easily synthesized catalyst for NO oxidation in industry.

## 2. Materials and Methods

### 2.1. Catalyst Preparation

HACFA was obtained from Beijing Energy Holding Co., Ltd., China. It was screened using a 100-mesh sieve. The composition of HACFA is summarized in Table 1. In this study, the Al_2_O_3_ content in HACFA was 46.62 wt% and the Al/Si ratio was 1.09. Analytical-grade H_2_SO_4_ and NaOH were obtained from Beijing Chemical Works (Beijing, China). A 50 wt% Mn(NO_3_)_2_ solution was supplied by Shanghai Macklin Biochemical Co., Ltd., China. All the solutions were prepared or diluted with deionized water. The following gases were supplied by Langfang Langwei Gas Co., Ltd., Langfang, China: N_2_ (99.99 vol%), O_2_ (99.99 vol%), and NO (99.99 vol%).

Based on a previous study, the mullite catalyst support was prepared via the acid–alkali combined extraction method [25]. In a hydrothermal reaction process, the HACFA was first treated in an H_2_SO_4_ solution (1.5 mol/L; liquid/solid ratio 7:1) at 85 °C for 90 min. Subsequently, it was immersed in the NaOH solution (200 g/L; liquid/solid ratio 15:1) at 95 °C for 150 min. The entire process was conducted using a hydrothermal reactor, which resulted in the production of MA support containing mullite whiskers. In the preparation of mullite catalyst support, impurities such as Fe_2_O_3_, TiO_2_ and CaO were removed from HACFA.

**Figure 1:**
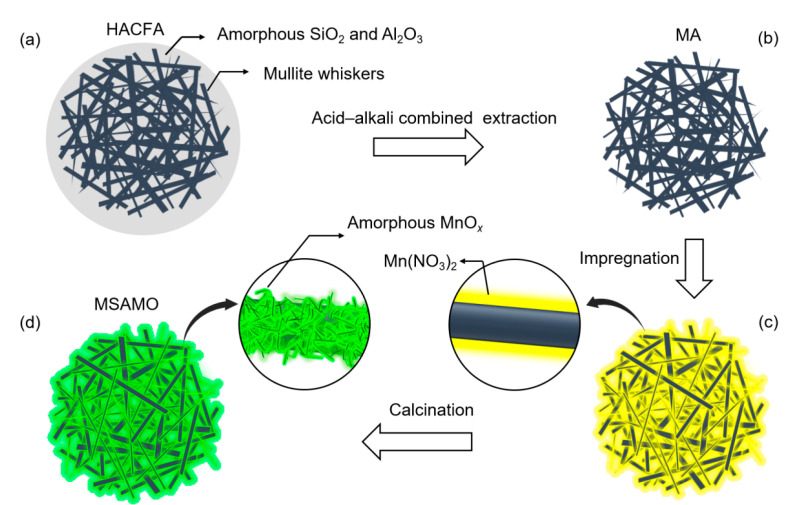
Schematic synthesis of MSAMO catalyst. (**a**) HACFA, (**b**) MA, (**c**) precursor of MSAMO, and (**d**) MSAMO.

The MSAMO catalyst was prepared via impregnation and calcination. First, the Mn(NO_3_)_2_ precursor was dissolved in 200 mL of deionized water, and 10 g of MA support powder was added and stirred for 1 h. Subsequently, the samples were evaporated in a rotary evaporator at 60 °C until they became dry and were calcined in a furnace under air atmosphere at varying temperatures. At 550 °C, the crystalline structure of MnO_x_ changed from amorphous phase to crystal phase [20]. Therefore, 500 °C was selected for calcination. Finally, the MSAMO catalysts were removed and cooled to room temperature after the calcination process. The schematic synthesis of MSAMO catalyst is shown in Figure 1.

### 2.2. Catalyst Characterization

X-ray diffraction (XRD, PANalytical, Alemlo, The Netherlands, X’pert PROMPD) was performed to analyze the crystal structures and phase compositions of the samples. Surface morphologies were observed via scanning electron microscopy (SEM, JEOL, Tokyo, Japan, JSM-7610F). X-ray photoelectron spectroscopy (XPS, Thermo Fisher Scientific, Waltham, MA, USA, ESCALAB 250) was employed to determine the relative contents of active components with different valence states. Sample pore-size distribution was characterized by a specific surface-area and pore-size analyzer with the Brunauer-Emmett-Teller (BET) method (Micromeritics, ASAP 2020HD88). Catalytic oxidation activities of the samples were analyzed via H_2_ temperature-programmed reduction (H_2_-TPR) using an automatic physicochemical adsorption instrument (Micromeritics, AutoChem II2920). The samples were reduced in gas containing 5 vol% H_2_/N_2_ flowing at 50 mL/min. The temperature and heating rate were set at 100–800 °C and 10 °C/min, respectively. In situ DRIFTS (Thermo, Nicolet 6700) was implemented to investigate the surface species involved in the catalytic oxidation of NO over the MSAMO catalyst.

### 2.3. Evaluation Method of Catalyst Activity

**Figure 2:**
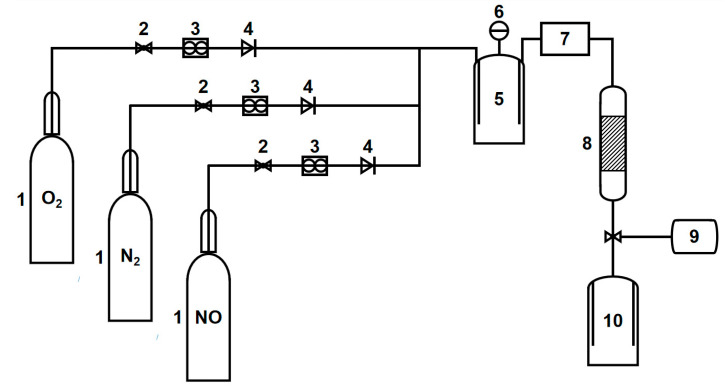
Schematic of catalyst activity evaluation device. Components include a (1) gas cylinder; (2) pressure-reducing valve; (3) flowmeter; (4) check valve; (5) mixing tank; (6) pressure gauge; (7) preheating furnace; (8) catalytic reactor; (9) gas analyzer; and (10) tail gas recovery tank.

A customized device consisting of three parts (gas distribution, reaction, and detection systems) was designed to evaluate the MSAMO catalyst activity, as shown in Figure 2. The catalytic reactor equipped with heating equipment had a diameter and height of 2.6 and 40 cm, respectively. The NO concentration at the reactor inlet was in the range of 500–1000 ppm, and the concentration of oxygen at the inlet was in the range of 3–10 vol%. Additionally, N_2_ was used as the equilibrium gas, conforming to the outlet gas concentration of a practical coal-fired boiler flue gas. The NO concentration at the reactor outlet was measured using a gas analyzer.

The NO conversion rate, *R*, is calculated using Equation (1):

(1) $$R=\frac{C_{i}-C_{o}}{C_{i}}\times 100\%$$

where *C*_i_ and *C*_o_ (ppm) denote the inlet and outlet NO concentrations in the flue gas of the catalytic reactor.

## 3. Results and Discussion

### 3.1. Phase Analysis

**Figure 3:**
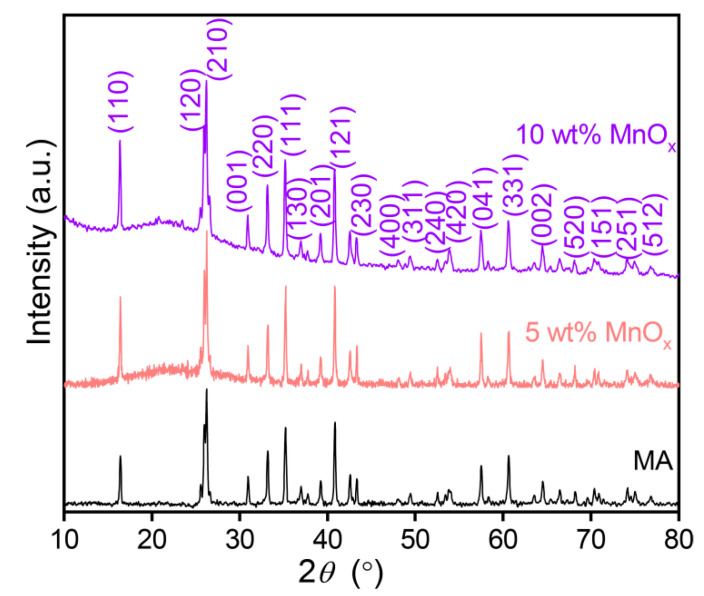
XRD patterns of MA and MSAMO catalysts with different MnO_x_ contents.

The XRD patterns of the MSAMO catalysts with 5 wt% and 10 wt% MnO_x_ (nominal composition) calcined at 500 °C for 4 h are shown in Figure 3. The catalyst support consisted of mullite (JCPDS#82-1237). Following the loading process, a steamed bun peak appeared in the range of 15–35° for the MSAMO catalyst, indicating that the MnO_x_ catalyst on the surface of the mullite supports was amorphous. The Rietveld method was used to calculate the content of amorphous MnO_x_, which was found to be 6.1 wt%, slightly higher than the nominal composition. The result can be attributed to the MnO_x_ encapsulation of MA particles.

### 3.2. Microscopic Morphology Analysis

**Figure 4:**
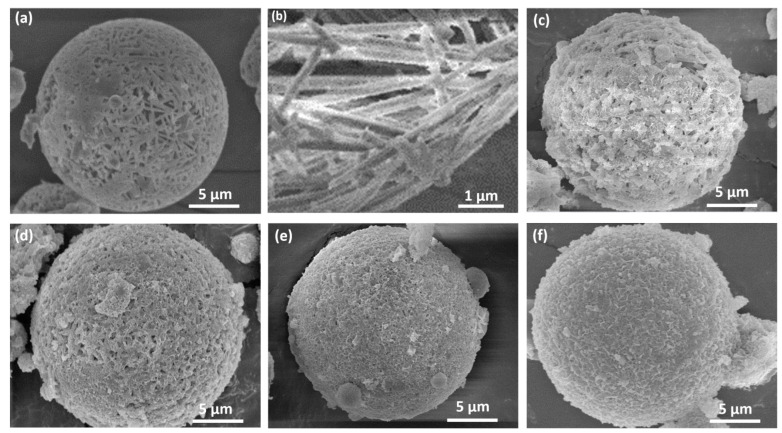
SEM images of MSAMO catalysts with varying MnO_x_ content. (**a**,**b**) 0 wt%, (**c**) 3 wt%, (**d**) 5 wt%, (**e**) 8 wt%, and (**f**) 10 wt%.

The effects of the amorphous MnO_x_ quantity on the MSAMO surface morphology are presented in Figure 4. As shown in Figure 4a,b, MA is a porous micron-sized spherical aggregate composed of mullite whiskers with a diameter of nearly 300 nm. The morphology of the MSAMO catalyst varies with the increase in amorphous MnO_x_. When the loading quantity was 3 wt%, mullite whiskers were observed in the support. The mullite whisker surface was covered with a layer of nano-flocculent MnO_x_. Furthermore, numerous pores on the surface of the MSAMO catalyst were observed, as shown in Figure 4c. As the amount of MnO_x_ increased, the coverage quality of amorphous MnO_x_ on the mullite whiskers improved. At a loading quantity of 5 wt%, a nano-flocculent layer was uniformly dispersed on the surface of the mullite whiskers without any apparent agglomeration. At this loading, the size of the pores on the surface of MSAMO decreased (Figure 4d). At a loading quantity of 8 wt%, most of the pores were blocked, and the agglomeration of amorphous MnO_x_ was observed on the surface of MSAMO, as shown in Figure 4e. When the loading quantity was increased to 10 wt%, the MSAMO catalyst was entirely covered by flocculent amorphous MnO_x_. The pores on the surface of MSAMO were blocked, and mullite whiskers were not observed. Additionally, amorphous MnO_x_ exhibits distinct agglomeration on the surface of MSAMO, as shown in Figure 4f.

**Figure 5:**
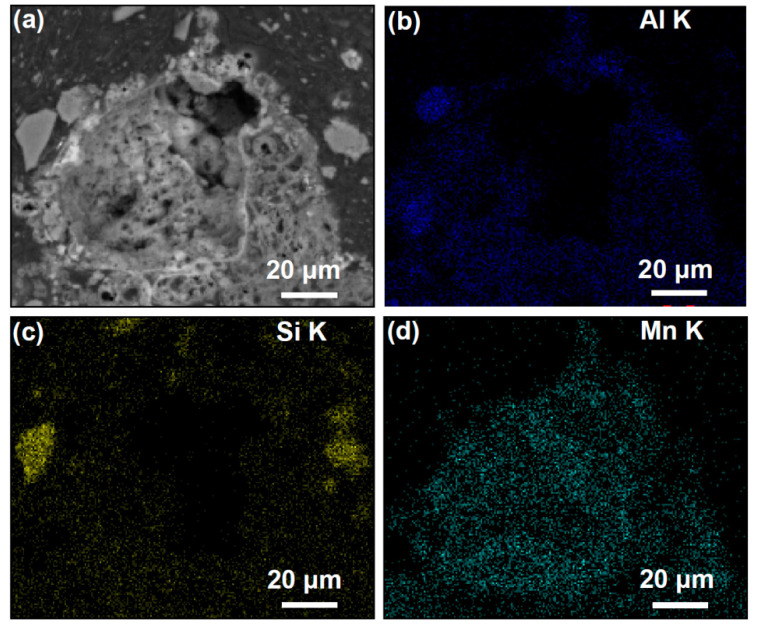
SEM–EDS image and element distributions of MSAMO catalyst with 5 wt% MnO_x_ (calcined at 500 °C for 4 h). (**a**) SEM image, (**b**) elemental mapping of Al, (**c**) elemental mapping of Si, and (**d**) elemental mapping of Mn.

The interior of MA support is composed of mullite whiskers, which contain numerous pores that offer multiple loading points for MnO_x_. To visualize the distribution of amorphous MnO_x_ in the MSAMO catalyst more clearly, energy-dispersive X-ray spectrometry (EDS) was performed on the profile of the MSAMO catalyst containing 5 wt% MnO_x_. The elemental distribution within the sample is presented in Figure 5. As seen in Figure 5a, the MSAMO catalyst has numerous internal pores. Additionally, aluminum and silicon were distinctly detected in the sample (Figure 5b,c). Moreover, manganese is evenly distributed throughout the surface and interior of the catalyst (Figure 5d). These results demonstrate that amorphous MnO_x_ enters the interior of the support through the pores and is evenly dispersed on the inner surface of MSAMO. Hence, when the MnO_x_ loading amount is 5 wt%, the MSAMO catalyst contains numerous internal pores and exhibits a highly effective loading capacity for MnO_x_.

### 3.3. XPS Analysis of MSAMO Catalyst

**Figure 6:**
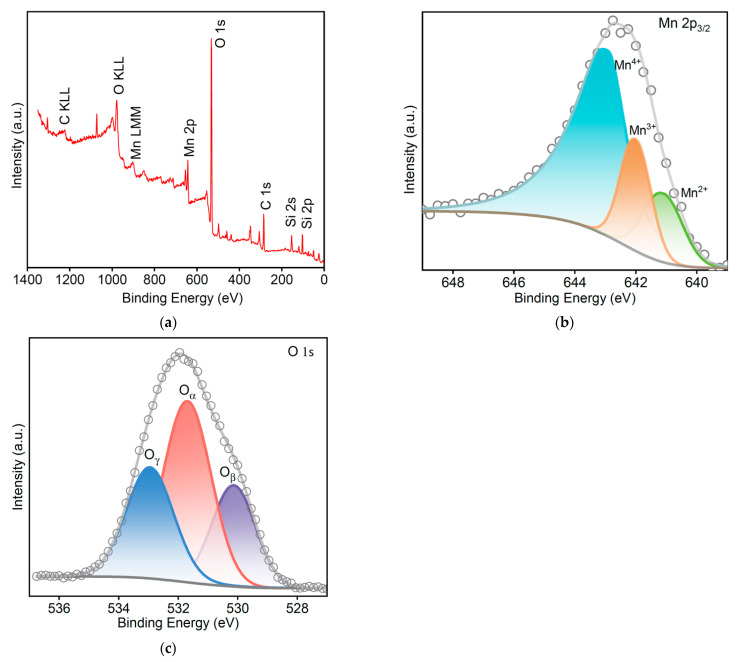
XPS core-level spectra of MSAMO catalyst with 5% MnO_x_: (**a**) survey wide scan spectrum, (**b**) Mn 2p_3/2,_ and (**c**) O 1s.

The valence states of manganese and oxygen in amorphous MnO_x_ were examined via XPS. The signal intensity ratios of Mn 2p_3/2_ systematically vary among the different oxides of manganese. This variation can be used to distinguish the different oxide species present in MnO_x_ [26]. The XPS wide scan survey spectrum from the 5 wt% MnO_x_ MSAMO catalyst is shown in Figure 6a. Based on the deconvolution of the XPS spectra, the Mn 2p_3/2_ peak is found to comprise three peaks corresponding to three manganese states: Mn^4+^, Mn^3+^, and Mn^2+^, at 643.5, 642.6, and 641.4 eV, respectively (Figure 6b), indicating that manganese exists in the mixed-valence state. This result is consistent with a previous report [27]. The fitted results show that MnO_x_ is composed of 66.1% Mn^4+^, 17.3% Mn^3+^, and 16.6% Mn^2+^. The Mn^4+^ content was higher than those of Mn^3+^ and Mn^2+^, which can be attributed to the oxidation of NO_3_^−^ during the high-temperature calcination process, leading to differences in the local oxidation atmosphere. More catalytic activity is provided by Mn^4+^ [19]; it is considered one of the most important factors for catalytic NO oxidation over MnO_x_ [28].

The O1s peak of the MSAMO catalyst was asymmetric. Based on the deconvolution result, the O1s peak comprises three peaks corresponding to: surface absorbed oxygen (O_α_), lattice oxygen O^2−^ (O_β_), and chemisorbed water molecules (O_γ_); they are located at 531.6, 530.0, and 532.8 eV, respectively (Figure 6c). This result is consistent with the data published by Zhang et al. [29]. The fitted results were 55.1% O_α_, 32.2% O_β_, and 12.7% O_γ_ in the MSAMO catalyst. Furthermore, O_α_ is an active substance that can promote the oxidation of NO into NO_2_ because it has higher mobility than O_β_, and it, therefore, plays a crucial role in catalytic oxidation.

### 3.4. Effect of MnO_x_ Quantity on NO Conversion Rate

**Figure 7:**
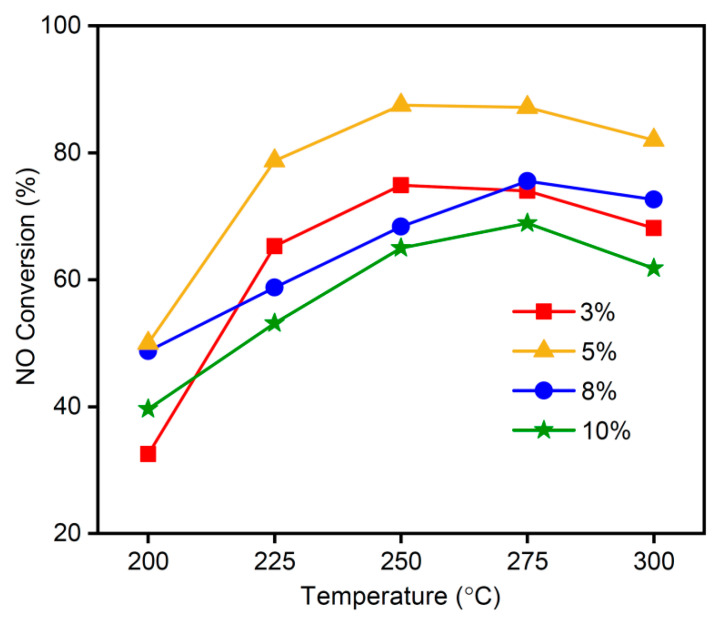
NO conversion rate of MSAMO catalysts with varying MnO_x_ quantities.

Figure 7 shows the influence of the MnO_x_ loading quantity on the MSAMO catalysts at different reaction temperatures. These sample materials were calcined at 500 °C for 4 h, and the reaction conditions for the catalytic conversion of NO were: a space velocity of 12,000/h; 800 ppm NO; 8 vol% O_2_; and N_2_ as a balance gas. When the MnO_x_ loading quantity was 5 wt%, the NO conversion rate was the highest for MSAMO catalysts at the same temperature, thereby showing the best catalytic performance. At a constant loading of MnO_x_, the NO conversion rate first increased and then decreased as temperature increased. The optimum reaction temperature range was in the range of 250–275 °C. When the loading amount exceeded 5 wt%, the conversion rate of NO began to decrease over the entire temperature range. For the MSAMO catalyst with 5 wt% MnO_x_, the maximum NO conversion rate was 88% at 250 °C. In this study, the optimal loading quantity of MnO_x_ was considerably lower than that reported in a previous study [30]. However, the maximum conversion rate of NO over the MSAMO catalyst was equivalent to that of MnO_x_ (0.3)/TiO_2_ (89% at 250 °C at a space velocity of 25,000/h) [30]. Additionally, the cost of support is lower in our study.

### 3.5. Effect of Calcination Time on NO Conversion Rate

**Figure 8:**
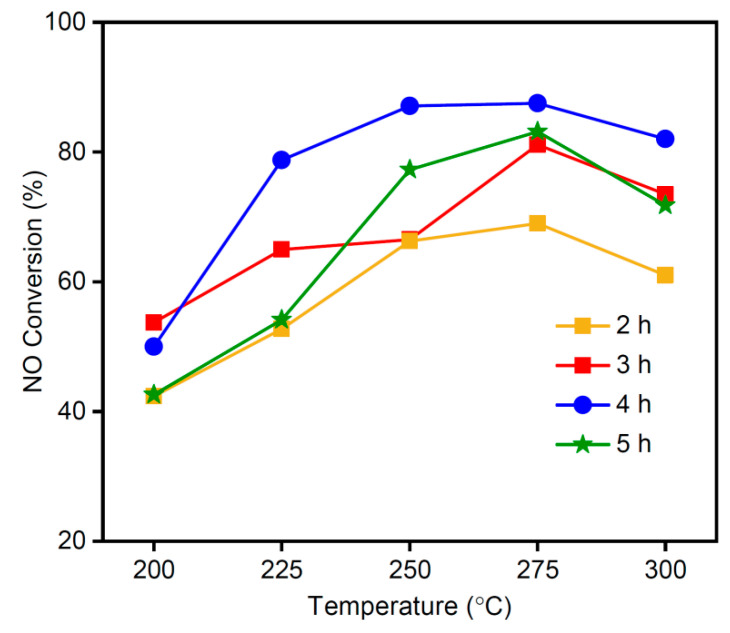
NO conversion rate of MSAMO catalysts calcined at 500 °C for four durations.

Figure 8 shows the NO conversion of the MSAMO catalyst (5 wt% MnO_x_) calcined at 500 °C for different durations. This trial was otherwise performed under the same conditions as those in Figure 7. The MSAMO catalyst that was calcined for 2 h and exhibited the lowest NO conversion rate in the lower temperature range (200–300 °C). As the calcination time increased, so did the NO conversion rate. When the calcination time was 4 h, the NO conversion rate reached a maximum value. If the calcination time is too short, then the decomposition of the precursor is incomplete, hindering catalytic efficiency. If the calcination time is exceedingly long, however, the existing form of the active substance can change in such a way as to reduce its activity. In the catalytic reaction temperature range of 225–300 °C, the NO conversion rate for the MSAMO sample calcined for 4 h exceeded 80%. The MSAMO catalyst material used in all subsequent experiments, therefore, comprises a MnO_x_ loading quantity of 5 wt% and a calcination temperature of 500 °C for 4 h. The NO conversion of the catalyst remained almost unchanged for 10 h at 250 °C. Therefore, the catalyst shows good stability.

### 3.6. Effect of Oxygen Content on NO Conversion Rate

**Figure 9:**
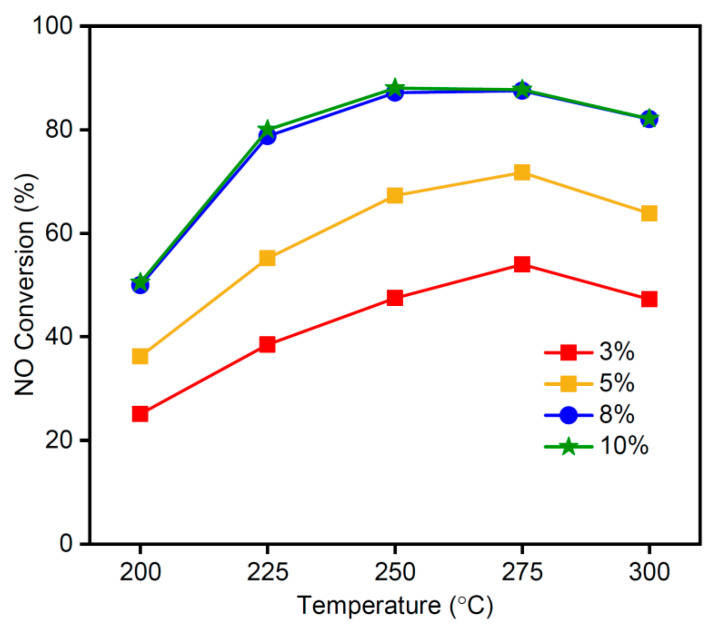
Effect of oxygen content on NO conversion rate for MSAMO catalysts.

The effect of oxygen content on the catalytic performance of MSAMO is shown in Figure 9. Experimental conditions were the same as previously used apart from the oxygen concentration, which was varied by 3–10 vol%. When the oxygen content was 3 vol%, NO conversion remained below 54%. The NO conversion rate significantly increased with the oxygen content. When the oxygen content was 8 vol%, NO oxidation tended to be stable with a conversion rate as high as approximately 88% at 250 °C. The catalytic oxidation activity of NO over the MSAMO catalyst was higher than that of an amorphous MnO_x_–CeO_2_ catalyst from the literature (60% NO conversion at 250 °C at a space velocity of 432,000/h) prepared via the co-precipitation method [31]. When the oxygen content was 10 vol%, the NO conversion rate did not change considerably. The amount of oxygen adsorbed on the catalyst increased with increasing oxygen content, promoting the catalytic oxidation of NO. However, when the oxygen content reaches 8 vol%, the surface tends to be saturated, so any change in the NO conversion rate is negligible. Generally, the oxygen content range in the flue gas of coal-fired boilers is 8–10 vol% [32]. Within this concentration range, the oxygen content was adequate for catalytic NO oxidation over MSAMO.

### 3.7. Effect of NO Concentration on NO Conversion Rate

**Figure 10:**
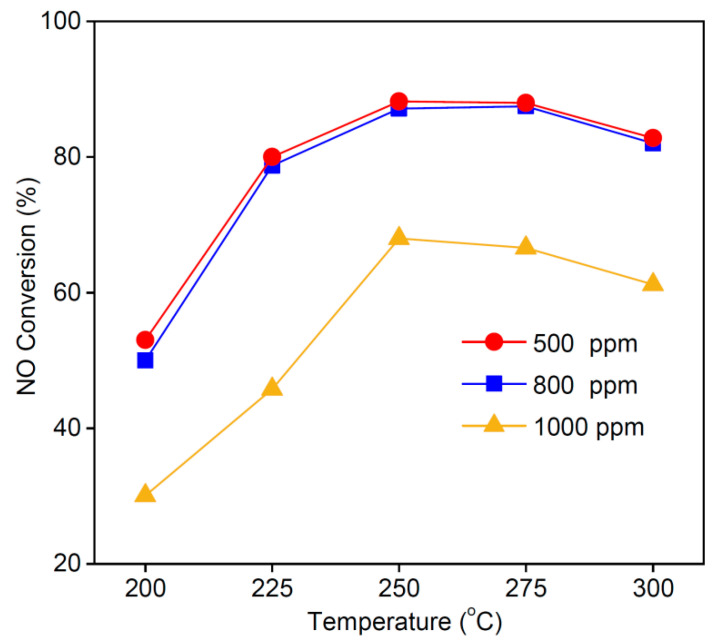
Effect of NO concentration on NO conversion rate of MSAMO catalyst.

We examined the effect of NO flue gas concentration on the catalyst conversion rate. The NO concentration range in the flue gas of coal-fired boilers is generally 200–1000 ppm. In this study, the NO inlet concentration was set to 500–1000 ppm, and the oxygen concentration was maintained at 8 vol%. Figure 10 shows the effect of different NO concentrations on the NO conversion rate of the MSAMO catalyst. The conversion rate did not change significantly when the concentration was increased from 500 to 800 ppm. However, the NO conversion rate decreased when the NO concentration was increased to 1000 ppm. The NO concentration in flue gas has an important effect on the catalytic performance of the MSAMO catalyst. If NO concentration is exceedingly high, NO and O_2_ will compete for active sites on the amorphous MnO_x_, leading to a reduction in both O_α_ and NO conversion. According to the equilibrium reaction equation, 2NO + O_2_ ⇔ NO_2_, the concentration of generated NO_2_ increases with the increase in NO concentration at the inlet, hindering the reaction progress. Consequently, when NO concentration at the inlet exceeded 800 ppm, the catalytic oxidation rate of NO over MSAMO began to drop. Therefore, 800 ppm NO was chosen as the optimal quantity for the flue gas used in this work.

### 3.8. Effect of Space Velocity on NO Conversion Rate

**Figure 11:**
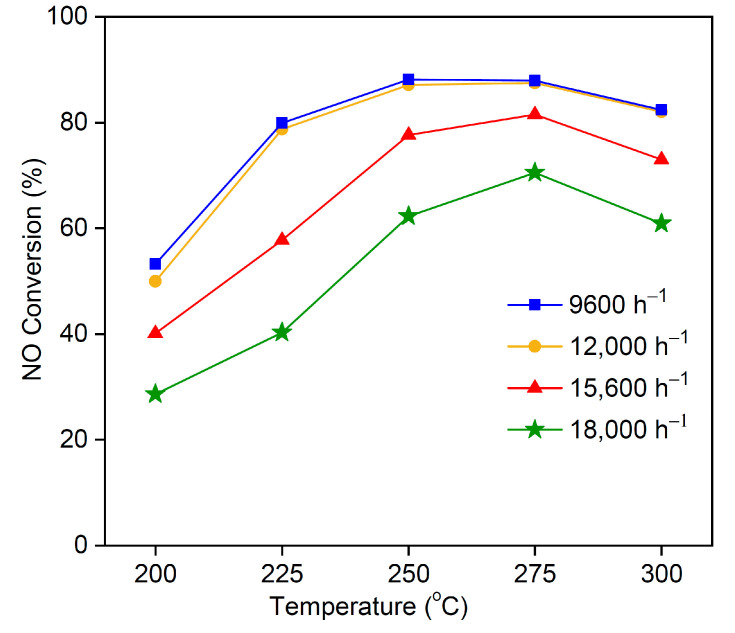
Effect of space-velocity on NO conversion rate over 5 wt% MSAMO catalyst at different temperatures.

The space velocity of flue gas varies in practice. An optimized gas mixture was passed through the catalytic reactor at different space velocities. Figure 11 shows the effect of space velocity on the NO conversion by MSAMO catalyst (5 wt% MnO_x_) at different temperatures. The NO conversion rate decreased as the space velocity increased at a constant temperature. Therefore, the space velocity has a negative impact on the NO conversion rate of the catalyst. The NO conversion rate decreased slightly as space velocity increased from 9600 to 12,000/h, reaching a maximum of approximately 88% conversion at 250 °C. When the space velocity increased from 15,600 to 18,000/h, the NO conversion rate decreased significantly. Molecular residence time increases with decreasing space velocity. In other words, at higher space velocities, the time a reagent spends in the catalytic reactor is shorter–potentially insufficient. Thus, a lower space velocity is favorable for achieving a higher NO conversion rate, which is consistent with previous research [14].

### 3.9. H_2_-TPR Analysis

**Figure 12:**
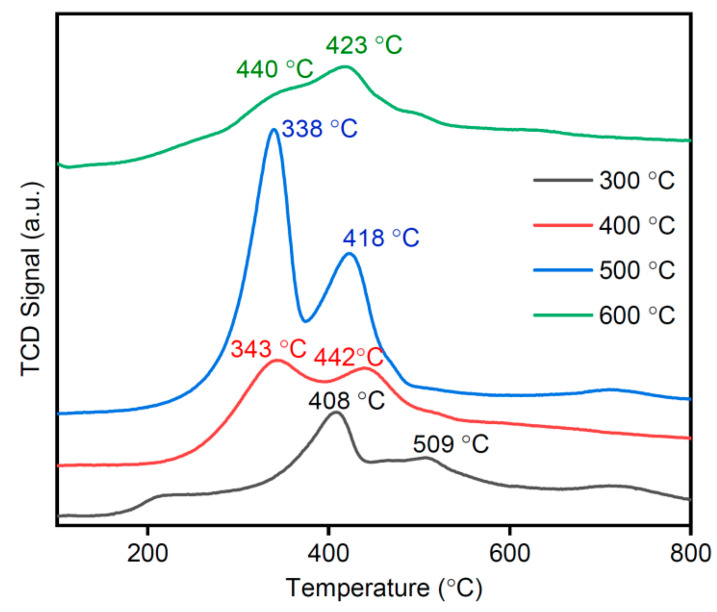
H_2_-TPR profiles of MSAMO catalysts at different calcination temperatures.

The ability of metal ions to transform from a high-valence state to a low-valence state can be detected by H_2_-TPR. This method was used to characterize the redox properties of the oxide, which is intimately related to the catalytic efficiency [33]. Figure 12 shows the H_2_-TPR profile (at a heating rate of 10 °C/min) of the optimized MSAMO samples that underwent calcination for 4 h at different temperatures. The MSAMO catalysts exhibited visible reduction peaks. Generally, MnO_x_ reduction undergoes a sequential process (MnO_2_→Mn_2_O_3_→MnO), and surface-adsorbed oxygen species are reduced in the H_2_ atmosphere [34]. These two mechanisms can be described by the following two equations.

(2) $${2\mathrm{MnO}}_{2}+H_{2}\to {\mathrm{Mn}}_{2}^{}O_{3}+H_{2}O$$

(3) $${\mathrm{Mn}}_{2}O_{3}+H_{2}\to 2\mathrm{MnO}+H_{2}O$$

According to Equations (2) and (3), the theoretical peak–area ratio of the two peaks in the H_2_-TPR profile is 2:1. Two reduction peaks were observed at 339 and 442 °C, representing the stepwise reductions Mn^4+^→Mn^3+^ and Mn^3+^→Mn^2+^, under H_2_ atmosphere from left to right, respectively [35]. This result is consistent with that of a previous study [20]. When the calcination temperature increased from 300 to 500 °C, the peak position shifted to low temperatures, and the peak–area significantly increased. This result indicates that high calcination temperatures facilitate manganese reduction in the catalyst. Compared to previous reports, the reduction-peak position of amorphous MnO_x_ is lower than that of its crystalline counterpart, and amorphous MnO_x_ exhibits better reducibility than crystalline MnO_x_ [20]. After calcination for 4 h at 500 °C, the best catalytic oxidation capacity was obtained. The peak–area ratio of the two reduction peaks at 339 and 442 °C is nearly 2:1, which is in agreement with the theoretical value. This is a typical characteristic of the reduction path of Mn^4+^ [9]. Hence, this result further demonstrates that the predominant active component in the MSAMO catalyst is Mn^4+^. For the MSAMO catalyst calcined at 600 °C, the peak area significantly decreased, indicating a decrease in reducibility. This decrease may be due to the decrease in O_β_, resulting in the deterioration of the redox ability of MnO_x_.

### 3.10. BET Measurement

**Figure 13:**
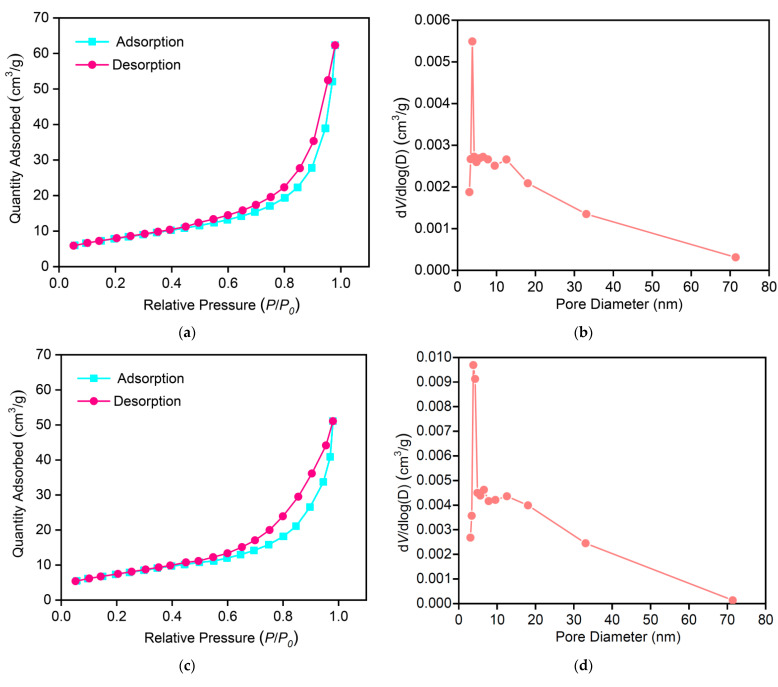
N_2_ adsorption and desorption isothermal curves and pore size distribution patterns of MSAMO catalyst calcined for 4 h at 500 °C. (**a**,**b**) 5 wt% MnO_x_, (**c**,**d**) 10 wt% MnO_x_.

Figure 13 shows the N_2_ adsorption and desorption isothermal curves and pore size distribution patterns of MSAMO catalysts calcined for 4 h at 500 °C. As shown in Figure 13, the macro-pores decreased with increasing the content of MnO_x_ from 5 wt% to 10 wt%, however, more meso-pores were developed. The specific surface area, average pore size, and pore volume of the sample with 5 wt% MnO_x_ were 30.189 m^2^/g, 17.965 nm, and 0.107 cm^3^/g, respectively. The sample presents a typical type IV adsorption isotherm loop in the physisorption isotherms at 198 °C due to the mesoporous structure [36], indicating an irregular pore structure [37], as shown in Figure 13a,c. At high pressure, the hysteresis loop indicates that adsorption is condensed in the capillary tubes in the macropores. Figure 13b shows that the catalyst exhibits a wide pore-size distribution, and the pore size is mainly concentrated at approximately 10 nm. The pore-size distribution curve of the sample shows a mixed structure of mesopores and few macropores, showing a relatively wide pore size distribution (Figure 13b). The sizes of NO and O_2_ molecules are approximately 0.317 and 0.346 nm, respectively [38], which are smaller than the pores of the sample. This large pore size exhibits satisfactory gas-diffusion ability, promotes chemical reaction, and improves the catalytic performance of the MSAMO material.

### 3.11. Mechanism of NO Catalytic Oxidation with MSAMO Catalyst

**Figure 14:**
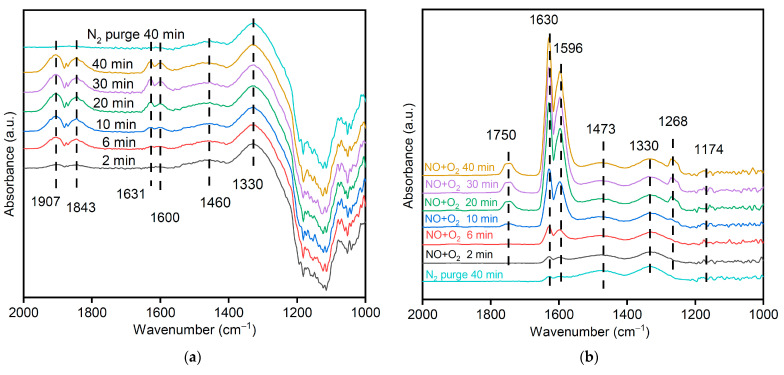
In situ DRIFTS spectra of MSAMO catalyst exposed to (**a**) 800 ppm NO and (**b**) 800 ppm NO + 8 vol% O_2_ at 250 °C with respect to time.

An in situ DRIFTS analysis was conducted to investigate the reaction characteristics of NO and O_2_ on the surface of MSAMO catalyst (Figure 14). The DRIFTS spectra of NO adsorption products on the MSAMO catalyst surface were recorded at different time intervals during nitrogen purging at 250 °C, as shown in Figure 14a. Absorption peaks were detected at 1907, 1843, 1631, 1600, 1460, and 1330/cm after NO was introduced. The peaks at 1907 and 1843 cm^−1^ correspond to the nitroso functional group [10]. The formation of nitroso groups is due to the coordination of the NO molecule with a Lewis acid (metal site) through nitrogen atoms, which results in a partial transfer of charge from the 5σ orbital and a strengthening of the N–O bond. The peaks observed at 1631 and 1600/cm correspond to the vibration absorption–peaks of bridge NO^3−^ [39]. These peaks show an initial increase, indicating that NO^3−^ can readily decompose. The absorption peak between 1500–1460/cm corresponds to the vibration peak of the chelated nitro functional group. Chelated nitro groups are reaction intermediates in NO oxidation. The peak at 1330/cm corresponds to the vibration absorption peak of the adsorbed NO_3_^-^ caused by the presence of O_β_ in amorphous MnO_x_.

The spectra of the products on the catalyst surface at different time intervals after NO + O_2_ adsorption saturation are shown in Figure 14b. After NO + O_2_ was introduced, different absorption vibration peaks were detected (Figure 14b). The peak at 1750/cm corresponds to the vibration absorption peak of NO_2_ dimer (N_2_O_4_), which is formed because the catalyst provides reactive oxygen species [40]. The peaks at 1630, 1596, and 1174/cm correspond to the vibration absorption peaks of bridging NO^3−^. The peak height gradually increases over time, and the peak strength is significantly higher than the absorption peak, as shown in Figure 14a. Therefore, O_α_ can increase the adsorption of NO on the catalyst after the introduction of oxygen. The peaks at 1473, 1268, and 1330/cm correspond to the vibration peak of chelated nitro, monodentate NO^3−^, and adsorbed NO^3−^, respectively.

In addition to O_α_, O_β_ is an important factor in the catalytic oxidation of NO [41]. The addition of gaseous oxygen promotes NO adsorption. The catalytic oxidation of NO begins with its initial adsorption onto the amorphous MnO_x_ in the MSAMO catalysts, followed by oxidation. This leads to the formation of NO^3−^, which eventually decomposes into NO_2_.

## 4. Conclusions

In this study, high-alumina coal fly ash was used to prepare a microspherical aggregate support via an acid–alkali combined extraction method to remove amorphous silica and alumina. Mullite-supported amorphous MnO_x_ catalyst was prepared by impregnation and calcination at low temperatures using manganese nitrate as the precursor and microsphere aggregates as the catalyst support. The effects of different preparation and catalytic reaction conditions of NO over catalyst were investigated. Moreover, the mechanism of the catalytic oxidation of NO into NO_2_ over the catalyst was examined. The main conclusions are as follows. The acid–alkali combined extraction method can be used to remove amorphous silica and alumina from the coal fly ash to obtain microspherical aggregates. The mullite whiskers can be used as catalyst support. Amorphous MnO_x_ was deposited evenly on the surface of the mullite whiskers in the aggregate spheres after calcination for 4 h at 500 °C. The catalyst with 5 wt% MnO_x_ loading exhibited good catalytic activity. The NO conversion rate was as high as 88%. Manganese exists in a mixed-valence state in the amorphous MnO_x_, and Mn^4+^ can provide more active sites. The catalytic oxidation of NO commences with initial adsorption on MnO_x_ and subsequent NO conversion, which results in the formation of nitrate that decomposes into NO_2_.

## Figures and Tables

**Table 1:** Chemical composition of HACFA (wt%).

Oxides	Al_2_O_3_	SiO_2_	CaO	Fe_2_O_3_	TiO_2_	LOI
Content	46.62	42.81	3.44	2.75	2.04	2.34

## Data Availability

Data can be obtained from corresponding authors upon reasonable request.
